# Supplementary material for: Are carbon emissions trading and green financial instruments synergistic? -Comprehensive quantitative research based on content analysis
Source: PLoS One. 2024 Mar 7;19(3):e0298601. doi: 10.1371/journal.pone.0298601 (PMC10919601; doi:10.1371/journal.pone.0298601)
Supplement: S1 Data — (ZIP) [file pone.0298601.s001.zip › Data/地级市Z_F工作报告.zip!/╒π╜¡/╠¿╓▌╩╨/└┤╫╘╣1⁄2╓┌║┼í╛┬φ┐╦╩2╛▌═°í┐_2014.docx]

各位代表：

　　现在，我代表台州市人民政府，向大会报告政府工作，请予审议，并请市政协委员和其他列席人员提出意见。

　　一、2013年工作回顾

　　2013年是落实省委干好“一三五”、实现“四翻番”的第一年。市政府在市委的正确领导和市人大、市政协的有力监督下，扎实开展“以实干论英雄”工作竞赛活动，直面难题，心无旁骛，强势推进各项工作，经济社会发展取得新成绩。预计生产总值增长8%，地方财政收入增长12.4%，固定资产投资增长19%，城镇居民人均可支配收入增长10%，农村居民人均纯收入增长10.5%，城镇登记失业率3.4%，人口自然增长率5.5%。，单位生产总值能耗降低率、主要污染物减排目标完成。

　　一年来，我们主要做了六方面工作：

　　(一)致力重大项目促进有效投资。集中精力推进重大项目，固定资产投资增幅居全省前列。甬台温高速复线乐清湾跨海大桥开工，沿海快速通道建设接近尾声，椒江二桥、82省道复线、机场路改建完工，内环线、76省道复线南延加快建设。金台铁路立项，市域铁路、台州炼化一体化项目前期积极推进。吉利V汽车、巨科新型铝轮毂项目开工，豪情汽车迁建项目加快建设，利源重工一期、天台国际汽车用品采购中心一期项目基本竣工。头门港区疏港公路、跨海大桥进展顺利，大麦屿港区对台直航客货滚装码头启动建设。台州第二电厂、仙居抽水蓄能电站加快建设。栅岭汪排涝调蓄工程全面建设，洪家场浦排涝调蓄工程开工。推进沿海开发，台州湾集聚区东部新区加速基础设施建设，形成一批可承接项目落地的成熟区块。头门港新区成立。三山北涂围垦完成，担屿涂、洋市涂加快围垦。加强土地要素保障，消化批而未供土地3.3万亩，供应土地4.87万亩，增长42%。天台列入国家低丘缓坡综合开发利用试点。加强政银企合作，企业直接融资84.88亿元。回归工程成果明显，引进省外资金151.7亿元，增长39.1%。

　　(二)致力创新驱动推动转型升级。坚定不移实施创新驱动战略，经济质量和效益稳步提升。加强浙大台州研究院等公共创新平台建设，新增国家认定企业技术中心1家、高新技术企业38家，新增省级企业研究院7家、研发中心33家。继续实施“500精英计划”。推进工业强市建设，传统产业提升工程和战略性新兴产业培育工程取得新进展，与中国北车、中国开发性金融促进会和中交集团分别签署战略合作框架协议。金属资源再生产业基地成为国家“城市矿产”示范基地。

　　温岭鞋业列入国家级提升示范项目，中国通号集团万全信号有限公司落户温岭。积极实施“四换三名”工程，扎实推进个转企、小升规、规改股、股上市，个转企9579家、三年任务一年完成，小升规400家，新增上市企业1家、报会企业4家。国家质量强市示范城市通过验收。大力发展现代服务业，市区服务业十大重点区块加快建设，新增省级服务业集聚示范区2家。筹建村镇银行1家，成立小贷公司3家。举办首届网商节，成为国家电子商务示范城市创建单位。绿心生态区成为省级旅游度假区，大神仙居景区开业。浙台海洋产业投创中心、台绣文化创意设计产业园、现代创意园开工。黄岩模塑工业设计基地二期基本建成。市农副产品集配中心动工，市中心粮库、麦德龙项目努力推进。农贸市场改造提升三年任务完成。积极培育新的消费增长点，社会消费品零售总额增长13%。建筑业、房地产业保持平稳。积极发展现代农业，新增粮食生产功能区8.43万亩、省级现代农业综合区2个、主导产业示范区3个。与省农科院签订全面合作协议。培育股份合作农场25家、家庭农场307家，组建农民专业合作社联合社15家。仙居成为全国农技推广示范县。推进农民素质提升工程，培训农民5.27万人。完成六大渔港经济区规划编制，6个渔港主体工程完工。

　　(三)致力改革开放激发发展活力。不失时机深化改革，重点领域实现新突破。出台行政审批制度改革“1+7”文件，商事登记改革走在全省前列。椒江实行企业投资项目业主承诺制。天台在全省率先实施中介机构改革。加快省级小微企业金融服务改革创新试验区建设，市金融服务信用信息中心、信用保证基金运行中心成立，海峡两岸小微金融发展论坛成功举办。组建市公交集团，市区公交一体化实现突破，公交分担率较快提升。试行市级国有资本经营预算，市开发投资集团成立。推进县级公立医院和基层卫生综合改革，启动市级公立医院改革。事业单位分类改革稳步实施。临海省级农村金融改革试验区、温岭省级现代农业经营制度创新试验区建设取得成效，玉环列入全国村级公益事业一事一议财政奖补试点。三门大力调整乡镇区划，仙居大幅度撤并行政村。深化口岸大通关建设，台州港货物吞吐量、集装箱吞吐量分别增长5%、10.4%，台州口岸成为特种货物进口指定口岸并实现转关运输，反走私领域企业守法诚信体系建设经验在全国推广。实施外贸稳增长行动，自营出口增长7%左右。举办驻沪商务领事、非洲官员走进台州活动，对外交流不断拓宽。

　　(四)致力整治提升改变城乡面貌。乘势而上抓好专项工作，促进城市有机更新。“三改一拆”工作全省领先，拆违3005万平方米、“三改”2966万平方米，提前完成省定三年任务，拆出了发展空间，拆出了城市形象，拆出了政府公信。交通治堵进度居全省前列，加强堵点疏导，打通断头路，整理停车位，规范行车秩序，促进微循环。水环境整治强势推进，实施百项整治工程，完成市区10条河道综合治理，新建污水管线55.33公里。化工恶臭整治成效显著，椒江医化行业累计退出恶臭项目142个、26家医化企业退出或转产、7家保留企业完成整治提升，黄岩退出72个项目、关停13家，主城区告别恶臭。加大重污染行业整治，淘汰关停119家企业。“多城同创”工作全力冲刺，领导包干破百难，众志成城齐创建，马路市场整治、城中村改造、户外广告清理等难点有效突破，创建成效明显。“四边三化”、“双清”行动扎实推进。中心城市加快建设。推进市区融合区块开发，中央商务区台州银行等5幢大楼结顶，刚泰国际中心开工。商贸核心区、江北商务区、飞龙湖、环白云山等区块加快建设。市规划展示馆和博物馆、科技馆建成。台州客运中心南站迁建、黄岩综合客运枢纽站动工。市地理信息公共服务平台开通。美丽乡村建设全面推进，359个待整治村完成建设，34个省级农房改造示范村、100个美丽乡村精品村启动建设，9个村落入选中国传统村落保护名录，2个村镇入选中国最美村镇。新农村电气化市通过验收。生态市建设积极推进，成为全国绿化模范城市，仙居创建国家生态县、玉环和三门创建省级生态县通过验收，椒江、黄岩、仙居成为省级森林城市，天台列入全国生态文明建设试点，仙居列入省级水生态文明建设试点。长潭水库38米线以下移民拆迁完成。椒江、台州湾集聚区实施初始排污权有偿使用制度。

　　(五)致力民生改善促进社会和谐。千方百计办实事，民生福祉不断改善。加强就业和社会保障。城镇就业新增6.12万人，城乡居民养老保险、城镇职工医疗保险参保人数分别新增13.6万人、12.4万人，新农合参合率99.78%。保障性安居工程新开工1.51万套(户)、竣工6119套(户)。新增养老机构26家、床位5314张。成为省扶残助残爱心城市。低收入农户奔小康工程扎实推进。大力发展社会事业。学前教育三年行动计划顺利完成，基础教育内涵不断提升，职业教育现代化工程稳步推进，高等教育毛入学率51%。实施医疗资源“三千提增计划”，15个县乡联合住院分部启动建设，市恩泽医院、市第二人民医院即将竣工。加强食品药品监管体系建设，乡镇(街道)食品药品安全工作站全部设立，百万学生饮食放心工程扎实推进，食品安全九大攻坚试点项目积极开展。文化惠民工程深入实施，乡镇综合文化站和村级文化活动室实现全覆盖，建成村级文化礼堂168家。临海成为省公共文化服务体系示范区。市广播电视集团成立。浙江佛学院天台宗学院、浙江道学院获准筹建。竞技体育成绩喜人，在十二届全运会获得1金2银11铜、在世界体操锦标赛获得1金。人口计生优质服务提升年活动和双基工程深入开展，出生性别比继续下降。市档案馆晋升国家一级档案馆。统计、地方志、气象等工作不断加强，妇女、儿童、老干部、关心下一代、慈善等事业取得新成绩。加强和创新社会管理。“三严三治三强”工作扎实推进，社会治安防控体系不断健全，“六五”普法深入开展，社会稳定风险评估机制、矛盾纠纷大调解体系不断完善，信访工作进一步加强，安全生产三项控制指标实现下降。部分企业资金链风险得到妥善处置。流动人口服务管理网络体系逐步完善。“四网融合”便民服务体系建设全面推进。“智慧水务”启动建设，市防汛应急(森林消防)指挥中心开工，创建9个国家级和11个省级减灾示范社区，成功防御强台风、超强降雨。村级组织换届选举基本完成。援疆和对口帮扶四川藏区等工作积极开展。国防建设、国防教育、国防动员、民兵预备役和海防、人防等工作得到加强。

　　(六)致力改进作风强化自身建设。严格贯彻中央、省委和市委关于作风建设有关规定，开展正风肃纪专项行动，政风、行风建设得到加强。认真落实市人大及其常委会决议决定，自觉接受人大依法监督、政协民主监督，认真听取民主党派、人民团体和各界人士的意见建议，办理人大代表议案建议180件、政协提案367件，办结率100%。连续七年成为全省依法行政优秀单位。继续实施“三提工程”，市本级94.9%审批事项承诺办理时限达到省内最短，行政权力规范化电子政务平台加快建设。实施市级公车改革。推进预决算公开，加强财政支出绩效管理，市级“三公”经费下降19.3%，其中公务接待费下降36.4%。强化审计监督。完善廉政风险干预机制，“阳光工程”深入推进，腐败案件查办力度不断加大。

　　各位代表!一年的变化，诠释着台州发展的律动和轨迹;一年的努力，凝聚着全市人民的汗水和智慧。在此，我代表市政府，向全市人民和外来建设者，向人大代表、政协委员，向各民主党派、工商联、各人民团体和社会各界人士，向驻台部队官兵、在台省部属各单位，向关心和支持台州发展的海内外朋友们，表示衷心的感谢!

　　成绩来之不易，经验弥足珍贵。我们深深体会到，知难而进，攻坚克难，首要的是树立良好工作导向，以实干论英雄，以实绩促发展;重要的是善于把握工作大局，顺势而为，破难而进;关键的是保持科学工作态度，精心谋划，果断决策，善作善成;根本的是坚持以人为本，做人民群众所希望做的事，依靠人民群众推动事业发展。

我们也清醒地看到，经济社会发展中还存在不少困难和问题。主要是：经济稳中回升的基础还不够稳固，生产总值、进出口增速未能达到预期目标;结构性矛盾依然突出，转型升级任重道远;制约发展的观念、体制因素仍然存在，改革任务繁重;中心城市辐射带动能力不强，城乡统筹发展任务艰巨;社会发展水平相对滞后，关系群众切身利益的环境保护、食品安全、医疗卫生等方面还存在不少薄弱环节，生态修复、改善民生、维护稳定压力较大;政府职能转变仍需加快，服务意识、办事效率与群众期待还有不少差距，改进工作作风、加强廉政建设还需付出更大努力。我们一定高度重视，坚持问题导向、底线思维，采取有力措施切实加以解决。

二、2014年目标任务

　　2014年，是贯彻党的十八届三中全会精神、全面深化改革的第一年，也是撤地设市20周年、掀开台州发展新篇章之年。面对改革重任，切实增强勇于担当、敢为人先的使命感。十八届三中全会对全面深化改革作出了新的重大部署，开启了新的改革窗口，开创了新的战略机遇，改革进入攻坚期和深水区。必须拿出舍我其谁的气魄、自我革命的勇气，抓铁有痕、踏石留印的狠劲，逢山开路、遇水架桥，将改革共识转变为改革红利。面对竞争挑战，切实增强居安思危、不进则退的危机感。当前全球经济进入深度转型调整期，城市化、资本化、金融化“新三驾马车”将促进经济转型，区域竞争日趋激烈，面临标兵渐远、追兵渐近的双重压力。必须思危求进，拉高标杆，将转型压力转变为创新动力，在激烈竞争中突出重围、赢得主动。面对重大机遇，切实增强只争朝夕、负重奋进的紧迫感。我国发展仍处于重要战略机遇期，改革、城镇化、人才“新三大红利”将支撑未来经济发展。浙江加快推进国家海洋经济发展示范区建设。台州自然禀赋山海兼利，发展空间得天独厚，民间投资充满活力，转型升级蓄势待发。必须着眼于连通沿海大通道、构筑沿海大平台、形成沿海经济带，将沿海地域优势转变为战略优势，借势发力，赶超发展。面对人民期盼，切实增强服务群众、造福于民的责任感。人民群众期盼富民强市、让发展成果惠及全市人民，期盼高效服务、切实感受作风转变带来新风和便捷，期盼良好生态环境、迎来碧水蓝天。必须将民生诉求转变为政府工作要求，倾心倾力，不辜负全市人民的重托。

　　今年政府工作总体要求：全面贯彻党的十八大和十八届三中全会精神，认真落实省委十三届四次全会和市四届三次党代会决策部署，坚持稳中求进、改革创新、实干争先，以深化改革为强大动力，以转型升级为核心任务，以生态文明为长远大计，以改善民生为根本目的，突出抓改革、促转型、治环境、惠民生，以优异成绩向撤地建市20周年献礼!

　　今年经济社会发展主要预期目标，综合考虑发展趋势和工作导向。一是实现更有质量的增长。以提高经济增长质量效益为中心，在稳增长中推动增长方式由要素驱动向创新驱动转变，构建内涵式发展新格局。生产总值增长8%，研究与试验发展经费支出增长14.8%，服务业占生产总值比重提高1个百分点。二是实现更有动力的增长。以推进城市化为引擎，以有效投资为支撑，以扩大需求为基础，增强发展内生动力。固定资产投资增长15%以上，社会消费品零售总额增长12%，外贸进出口增长5%。三是实现更有持续的增长。树立“绿水青山就是金山银山”理念，以“五水共治”为抓手强化生态环境保护，促进人与自然相和谐，经济社会发展与人口资源环境相协调。人口自然增长率控制在6%。以内，单位生产总值能耗下降1.5%，化学需氧量、氨氮、氮氧化物分别削减2%，二氧化硫削减1%。四是实现更有福利的增长。既做大财政“蛋糕”，确保地方财政收入增长8%，又分好财政“蛋糕”，确保新增财力向民生倾斜，提高发展的包容性。居民消费价格涨幅控制在3.5%以内，城镇居民人均可支配收入增长9%，农村居民人均纯收入增长9.5%，城镇登记失业率控制在4%以内。

　　为实现上述目标，重点做好以下八方面工作：

　　(一)着力激发各项改革红利，推动台州发展焕发新活力。

　　以更大的勇气和智慧深化改革，聚合正能量，激发新红利，增创台州科学发展体制机制新优势。

　　深化经济体制改革。围绕使市场在资源配置中起决定性作用，激发市场主体活力。落实非禁即入原则，建立“负面清单”，促进贸易和投资便利化。争创国家小微企业金融服务改革创新试验区，建立地方金融发展资金池，组建浙江(台州)小微金融研究院。深化临海金融创新示范市和农村金融改革试验区建设，做好路桥、临海、温岭省级民间融资管理创新试点。探索民间投资改革创新，争创国家民间投资改革创新示范区。推进国有资产监管全覆盖，规范国有企业管理人员薪酬水平。完善港口一体化发展机制。推进资源要素配置改革，坚持“以亩产论英雄”，建立按综合效益配置资源要素的导向机制。推开海域使用权招拍挂制度。完善资源有偿使用和生态补偿制度，推行节能量、碳排放权、排污权、水权交易制度。

　　加快城乡一体化和社会领域改革。围绕城乡发展一体化、增强社会活力，推动全社会共融共生。落实农民对承包地占有、使用、收益、流转及承包经营权抵押、担保权能。推进宅基地跨区域空间置换，稳步开展农民住房财产权抵押、担保、转让试点。加快村经济合作社股份制改革，探索开展农村集体经营性建设用地出让、租赁、入股试点。推进农民住房“立改套”。扶持集体经济薄弱村发展。大力发展山区经济。开展省级现代农业经营机制创新试点。完善农业保险制度。建立公共资源城乡均衡配置体制，促进城乡基本公共服务均等化。开展玉环户籍制度改革试点，有序推进农业转移人口市民化。探索都市区统筹协调发展机制，争取省都市区经济试点。加快市区供水一体化，适时推进水务体制改革。深化教育、卫生、文化体制改革，进一步发展民办学校、民办医院、民办文化。健全就业和社会保障体制，深化收入分配制度改革，创新社会治理体制。

　　推进行政体制改革。围绕政府职能转变，切实从“越位点”退出，把“缺位点”补上、“在位点”做好，以更有效的“管”促进更积极的“放”。深化行政审批制度改革，继续精简一批、下放一批、提速一批审批项目，逐步做到审批清单之外的事项由市场主体依法自行决定。建立全流程联合审批机制，完善全过程实时监督、行政问责、责任追究制度。实施新一轮政府机构改革，推进机构和职责整合，严格控制机构编制。支持玉环撤县设市。因地制宜推进乡镇、行政村撤并和撤村建居。探索适合小城镇发展特点的行政管理体制。加快事业单位分类改革，建立事业单位法人治理结构。改革预算管理制度，完善预算公开制度和绩效管理制度，加大专项资金整合力度。改进政府采购制度，健全政府向社会购买服务机制。强化市场秩序监管，完善信用约束机制，将有违规行为的市场主体列入经营异常的“黑名录”，使其一处违规、处处受限。

　　(二)着力实施创新驱动战略，推动转型发展取得新突破。

　　以最大的共识、最强的共振，创新驱动，加快转型，打造台州经济升级版。

　　提升自主创新能力。以“八倍增两提高”行动为抓手，加快建设创新型城市。推进浙大台州研究院、中科院台州应用技术研发与产业化中心等创新平台建设，加强与大院名校合作，谋划建设台州科创园。深化军民合作、校企对接、院士专家项目对接活动，培育网上技术市场。大力培育科技型企业，新增国家重点扶持高新技术企业42家、省级高新技术企业研发中心10家、省级科技型中小企业100家。大力培养高素质企业家队伍。深入实施“500精英计划”，加快人才平台建设，加大高层次创新人才引进力度。深化国家知识产权示范城市建设。加快培育创投基金，建立科技人才发展基金和科技金融服务中心。

　　打造工业主导优势。把工业发展摆上突出战略地位，加快建设工业强市。扎实推进36个县域块状经济向现代产业集群转型升级。完成工业主导行业转型升级规划编制，重点抓好医药、汽摩行业转型提升，推动医药产业绿色发展，培育汽车产业集群。深入实施战略性新兴产业培育工程，加快建设百家重点企业、百项重点项目。全面实施“四换三名”工程，鼓励企业立足实体经济扩大投资，加大现代技术改造，推进工业化和信息化深度融合，力争工业性投入增长15%以上。深入实施大企业培育工程，推进个转企、小升规、规改股、股上市，新增规上企业400家。积极发展现代建筑业。深化质量强市示范城市建设，实施品牌和标准化战略，创建国家级出口机动车和省级出口阀门质量安全示范区。

　　培育服务业支柱产业。促进服务业发展提速、比重提高、水平提升。大力发展金融业，引导地方城市商业银行做专做强，加快村镇银行发展，推进农村合作金融机构股份制改造，规范发展小额贷款公司和农村资金互助社，大力发展农村普惠金融。推动现代物流业发展，推进市物流发展交易中心、市粮食批发市场和中心粮库建设。大力实施旅游提升工程，争创国家5A级景区和国家级生态旅游示范区，加快建设天台山、仙居永安溪、临海古城、三门蛇蟠岛等旅游休闲集聚区，推进绿心风景旅游度假区、九子山风景旅游度假区、温岭龙门湖旅游综合体和石塘半岛旅游区建设。推进万达广场等大型商贸综合体、专业市场和配送中心建设。开展国家电子商务示范城市建设，加快建设电子商务园区。促进会展业发展。推动房地产业转型发展。大力发展健康、养老等民生服务业，吸引民间资本参与建设健康综合体，启动绿心生态养老文化村建设，把服务老年人“夕阳红”事业打造成蓬勃发展的朝阳产业。

　　巩固农业基础优势。加大强农惠农富农政策支持力度，让农业经营有效益，让农业成为有奔头的产业，让农民成为体面的职业。切实保障粮食安全和主要农产品有效供给，确保粮食播种面积194万亩、总产量74万吨。推进农业“两区”建设，加快建设现代农业综合体，新建粮食生产功能区7.34万亩，基本建成现代农业综合区3个、主导产业示范区5个。推进农业特色产业“三化”示范工程，实施与省农科院院地合作三年行动计划，加快创新农作制度重点乡镇和示范园区建设，大力发展现代种业，加快发展生态循环农业、都市农业、有机农业、休闲观光农业，提升发展农家乐。推行“肥药双控”，健全农产品质量安全监管体系。构建专业合作、供销合作、信用合作“三位一体”农村新型合作体系，着力发展股份合作农场、家庭农场，提升发展农民专业合作社，做大做强农业龙头企业，促进“两水一加”传统产业升级。积极鼓励和引导农业走出去。加强基层农业公共服务中心建设。推进农民素质提升工程，支持大学生从事现代农业，培养现代农业经营人才和新型职业农民。

　　(三)着力完善重大基础设施，推动区位条件实现新改变。

　　以砸锅卖铁的决心，打好基础设施建设攻坚战，为台州未来发展奠定基础。

　　完善综合交通体系建设。深入实施交通“211工程”，重点推进“一体两翼、一环两线”建设，确保交通投资超百亿。甬台温高速复线台州段全面启动，全线统筹，分段实施，加快建设。争取金台铁路及头门港支线、市域铁路、杭绍台高速、台金高速东延二期、台金高速市区连接线开工，力争签订机场迁建协议，深化市区现代有轨电车项目研究。确保沿海快速通道、104国道改建天台段通车，104国道黄岩段、35省道仙居段改建等10个项目开工。

　　加快能源、水利、信息设施建设。扎实推进三门核电一期建设，争取玉环华能三期项目核准开工。加快500KV三门核电、仙居抽水蓄能电站送出线路等输变电工程建设。开工建设市区天然气高压输配工程，推进甬台温天然气(油品)管网建设。加快洪家场浦、栅岭汪、金清新闸二期等排涝调蓄工程建设，开工建设方溪水库主体工程、东官河综合整治工程，争取朱溪水库、大田平原排涝一期工程动工，推进台州三期供水、温岭骨干河道排涝、东屏水库、青龙浦、玉环楠溪江引水工程前期。做好“智慧水务”试点，推进“智慧城市”建设。

　　强化资金、土地要素保障。探索组建国有资本运营公司，成立市金融投资有限公司，建立产业基金、城市发展基金、铁路建设基金。增强金融保障能力，确保新增贷款超过500亿元。充分利用多层次资本市场，拓展企业融资渠道，加快直接金融园区建设，争取直接融资100亿元。推进海涂围垦，做好十二塘、南海涂、北洋涂二期围垦前期。积极向上争取和科学合理配置用地指标，完善节约集约用地评估制度，大力实施城镇低效用地再开发，加快“三改一拆”拆后土地、批而未供土地、荒滩未利用地和低丘缓坡开发利用，完成造田造地新增耕地1.65万亩，落实农村土地综合整治1700亩，建设高标准基本农田25万亩。开展基本农田保护补偿机制试点，加强土地卫片执法检查。

　　(四)着力构建重大发展平台，推动经济发展拓展新空间。

　　大手笔规划、大强度推进平台建设，切实增强发展带动力、支撑力、承载力。

　　全力推进台州湾集聚区建设。加快新区开发建设，推进路网、水网、绿网、电网等基础设施工程，推动住宅、教育、商业等公共服务设施建设，东部新区动工建设月湖绿岛区块和吹填二期工程，加快住宅小区邻里中心、商业服务中心建设，力争台州湾湿地公园开工，做好台州湾观光农业产业园和体育公园前期。创建省级高新技术产业园区，加快吉利V汽车、巨科新型铝轮毂项目建设，开工建设广汽吉奥项目，推进台州炼化一体化项目前期，提升金属资源再生产业基地发展水平。

　　积极打造蓝色经济优势板块。大力发展海洋经济，加快建设海洋强市。开展海洋功能区划修编，完成台州港总体规划修订。实施临港工业带动型、功能差异化、投资多元化、开发一体化发展战略，加快港口建设和整合，完善港口集疏运体系。加快头门港新区建设和口岸开放前期，完成一期码头泊位、后方场地建设，实现头门港区开港。推进大麦屿港区3万吨级码头建设和对台货运直航，开工建设228国道温岭泽国至大麦屿疏港公路。完成海门港区外贸码头改扩建和龙门港区多用途码头工程，推进健跳港区台州第二发电厂码头建设。加快临港装备制造基地、能源建材港航物流基地、海洋清洁能源产业基地建设，争取国家海水淡化产业园区项目。加快建设渔港经济区，推进避风港建设。加强大陈海洋旅游开发与保护示范岛建设。加快建设玉环海岛统筹发展试验区、海洋经济转型升级示范区和浙台(玉环)经贸合作区。推进三门湾次经济圈建设。

　　努力推动开发区提档升级。提升省级开发区(工业园区)承载能力、产业层次、产出比重和项目集聚程度，台州经济开发区努力创建国家级高新技术产业园区，支持三门等创建省级经济开发区。

　　(五)着力推进新型城市化，推动市域经济跨上新水平。

　　坚持“四化”同步，推进以人为核心的新型城市化，推动县域经济向市域经济转变。

　　优化城市空间布局。在构建台州城市群框架下，谋划海湾型都市区建设。开展城市总体规划修改，启动公共设施布局规划编制。加强城市地下管线规划和管理，推进地下空间开发和利用。强化“大台州”理念，促进中心城市各组团相向建设、融合发展。中央商务区加快中央公园、天盛中心、刚泰国际中心建设，打造城市精品区、城市新名片。商贸核心区启动银泰综合体、心海生态文化园建设。绿心生态区加快飞龙湖、植物(雕塑)园建设，动工建设东山湖，打造城市会客厅和休闲好去处。高铁新区启动王林洋岛、东南国际采购物流中心建设。加快建设西商务区玉兰广场城市综合体。推进台州高等教育区块开发建设。加快星明工业小区改造和东环大道沿线开发，推动江滨公园西进。高起点规划椒北开发建设。推进江北商务区、委羽山新区、新前城市新区和模具新城建设，启动江口区块开发。加强市区西部山区保护开发。推进环中央山、市场园区、新城区块建设。完善城市路网，建成台州大道三期，加快内环线、市府大道东延、聚海大道建设，开工建设桐江大道。加强城区山体保护、林相改造和城市公园建设，把城市建在自然中。注重挖掘保护历史文化遗迹，让居民记得住乡愁。按照副中心城市的定位，推进临海城市新区建设和古城保护开发，构建“一城五组团”城市格局;推进温岭东部新区、城市新区、铁路新区三大板块开发，建设现代化中等城市。推动玉环环漩门湾海岛新城建设，打造海上都市。深化天台“小县大城”战略，以始丰湖公园建设带动城市功能提升。以建设国际旅游目的地城市为载体，打造美丽仙居。加快三门“三港三城”建设，打造海洋强县。实施新一轮小城市试点三年行动计划，推动中心镇改革发展和小城市培育。

　　提高城市管理水平。深入实施“多城同创”，建立长效管理机制，深化市容环境综合整治，有序推进各项必备工程建设，加强市民素质教育，争取成为国家卫生城市、国家环保模范城市、全国文明城市提名城市。加大“三改一拆”力度，开展无违建县、乡村创建活动，抓好连片综合整治，带动重点区块改造提升。深入开展城市交通治堵，加快停车设施建设，优化市区公交、慢行等交通循环系统。加强社区建设，完善社区功能。建立“大城管”机制，提高城管执法和服务水平，推动城市高效、精细、智慧管理。

　　推进美丽乡村建设。深化“百村示范、千村整治”工程，推进美丽乡村“四级联创”，深入开展“双清”行动，推动农村环卫保洁常态化，集全民之力打赢农村生活污水治理攻坚战，启动20个省级农房改造示范村建设，做好9个省级重点历史文化村落保护利用，建成20个精品村，全面提升美丽乡村建设水平，让农村成为安居乐业的美丽家园。

　　(六)着力拓展市场需求，推动对外开放迈出新步伐。

　　以更加开放的视野、更加开明的姿态，实施更加主动的开放战略。

　　深化对外开放。落实外经贸促进政策，加强出口信用保险和信保融资应用，拓展经贸活动平台。完善出口基地公共服务平台，推进电子口岸建设，争取设立保税物流园区等海关特殊监管场所。推动跨境电子商务发展。推进海外台州商品分销中心、展示中心建设，拓展新兴市场，促进加工贸易、服务贸易发展。引导企业扩大先进技术设备、关键零部件和重要能源原材料进口。鼓励优势企业走出去投资建立经贸合作区，开展跨国并购和国际技术合作。完善招大、引强、选优机制，大力引进世界500强企业、央企、军企。确保实际利用外资3.5亿美元。

　　努力扩大内需。构筑现代商贸流通体系，加快专业市场转型升级，提升市场综合服务功能。健全农村现代流通网络，提高城镇二级市场消费能力，扩大大众消费。开展名品进名店、品牌下乡，组织工商对接、农商对接，推进流通领域品牌体系建设。发展网络、信用、服务等新型消费方式，积极培育信息、健康、养老、社区服务等消费增长点。

　　加强区域合作。积极对接上海自贸区，深化与台湾交流合作，争取成为海峡两岸“车辆互通”试点、开辟首个浙台两岸自驾游航线。大陈、天台争取创建国家级海峡两岸交流基地。营造重商亲商爱商氛围，加强异地商会建设，推进“浙商回归”工程，引进省外资金145亿元。做好对口帮扶和对口支援工作。

(七)着力加快美丽台州建设，推动生态文明取得新成效。

　　以铁的手腕，坚持不懈推进环境整治和生态建设，让水变清、地变净、天更蓝、人民生活更健康。

　　加大环境综合治理力度。大力实施“五水共治”，完善跨区域综合治理机制，加强截污工程建设、生态补水研究、河道综合整治、环境联动执法，强化污水收集量、COD减排指标、河道交接断面水质变化、66条重点河道改善的考核，全面推行“河长制”，全力推进水环境综合整治。深化医化行业污染整治，巩固主城区告别化工恶臭成果，推进江口、临海川南、仙居城南等医化园区告别恶臭整治。加强大气复合污染防治，力争PM2.5优良天数比例75%以上。推进土壤污染治理。开展垃圾焚烧场净化升级改造。全面完成“四边三化”三年计划。强化环境监管和执法，建立生态环境损害责任终身追究制。

　　推进生态环境保护。实施主体功能区战略和环境功能区划，划定并严守生态红线，健全优化生态空间管理制度。加强饮用水源保护，实施长潭水库库区土地租用退耕还林，推进生态湿地建设。开展台州湾、乐清湾、三门湾污染整治，加强椒江流域保护，加快永安溪、始丰溪、灵江生态修复和保护，推进鉴洋湖、漩门湾国家级湿地公园保护开发。市区、临海、天台创建省级森林城市，临海创建国家环保模范城市，仙居争创国家公园。

　　强化节能减排攻坚。实施能源“双控”和污染物排放总量控制制度，严格环境准入制度，强化节能减排重点工程建设和管理。推进循环经济行动，推行清洁生产，完成医化、金属熔炼、造纸、印染等行业整治提升任务。建立工业企业环保一厂一档制度，初步建成“数字环保”监管体系。建立资源环境承载能力监测预警机制，强化节能减排约束性指标考核，对完不成年度目标任务的实施区域限批和一票否决。

　　(八)着力改善民生促进和谐，推动社会建设获得新进步。

　　多谋民生之利，多解民生之忧，让市民更幸福、城市更包容、社会更温暖。

　　完善就业和社会保障体系。健全城乡均等的公共就业创业服务体系，加强创业小额担保贷款服务，推进以高校毕业生为重点的青年就业和农村转移劳动力、城镇困难人员、退役军人就业。整合城乡居民基本养老、基本医疗保险制度，健全社会保障待遇确定和正常调整机制，完善社会保险五费合征机制，有序推进机关事业单位养老保险制度改革。健全城乡居民最低生活保障制度。完善社会救助、社会福利体系，发展慈善事业。支持残疾人事业发展，保障妇女儿童合法权益。推进社会养老服务体系建设，编制实施养老服务设施布点规划。规范和促进保障性住房建设、管理和运营。完善住房公积金提取、使用和监管机制。稳步提高最低工资标准。稳妥推进机关事业单位工资制度改革。完善企业工资集体协商制度，鼓励民营企业开展职工持股会等制度创新。做好国家级移民避险解困试点。深入推进扶贫计划，促进城乡低收入家庭和欠发达地区群众增收。

　　大力发展社会事业。优先发展教育事业。启动省教育现代化市创建，实施学前教育第二个三年行动计划，加强义务教育学校标准化建设，全面实施校长教师交流轮岗工作，利用信息化手段扩大优质教育资源覆盖面，特色化多样化发展高中段教育，推进高等教育内涵式发展，加快发展现代职业教育，推动发展特殊教育。落实考试招生制度改革。实施百万学生素质提升工程，全面推进素质教育。开工建设台州高级中学、电大新校区，基本建成台州学院椒江校区三期工程，加快台州经济开发区新学校建设。大力发展医疗卫生事业。启动卫生强市建设，推进卫生强县创建。加快公立医院改革，鼓励民营医院向规范化特色化高端化发展。深入实施医疗资源“三千提增计划”，深化基层医疗卫生综合改革，完善合理分级诊疗模式，加强中心镇卫生院建设，利用信息化手段促进优质医疗资源纵向流动。允许符合条件的民办医疗机构纳入医保定点范围。完善中医药事业发展政策和机制。做好疾病防控、卫生监督工作，加强精神卫生防治体系建设。开工建设市妇女儿童医院、市公共卫生中心、市立医院新院区、市第一人民医院新区。加快发展文化事业。加强公共文化服务体系建设，促进基本公共文化服务标准化、均等化，推动数字文化服务网络建设。深入实施文化惠民工程，推进农村文化礼堂建设。广泛开展群众性精神文明创建活动。实施文化产业发展工程，培育文化骨干企业，加强文化精品创作。完成广播电视有线网络一省一网整合。做好台州府城墙申报世界文化遗产工作，加强台州乱弹保护传承。推进体育强市创建，新增省体育强县2个。深化全民健身活动，重视老年体育工作，开工建设市少体校。夯实计生基层基础，稳妥实施单独两孩政策。加强统计基础建设，做好第三次经济普查。开展基本侨情调查。加强民族宗教、外事、对台事务工作，做好档案、地方志和气象工作。

　　加强社会治理创新。坚持系统治理、依法治理、综合治理、源头治理，改进社会治理方式。以平安村居、平安社区、平安家庭创建为载体，深化平安台州建设，健全平安创建工作机制。建立畅通有序的诉求表达、心理干预、矛盾调处、权益保障机制，深化人民调解、行政调解、司法调解联动衔接的大调解工作体系。完善社会稳定风险评估和突发事件应急处置机制，重视新媒体运用和管理，提高网络舆情研判导控处置能力。实行网上受理信访制度，健全涉法涉诉信访依法终结机制。完善司法救助和法律援助制度。优化外来人口结构。深化网格化管理、组团式服务，完善“四网融合”便民服务体系，加强乡镇(街道)社会服务管理中心规范化建设。抓好温岭基层治理现代化省级试点。推进社会组织健康有序发展，重视社会工作者队伍建设。加强社会治安综合治理，推进“三严三治三强”工作，完善立体化社会治安防控体系，依法严密防范和惩治违法犯罪活动。切实维护医疗秩序，努力构建和谐医患关系。推进殡葬改革，加强丧葬礼俗管理。完善食品药品城乡一体化监管体系，实施最严格的食品药品安全监管制度，建立食品原产地可追溯和质量标识制度，确保人民群众“舌尖上的安全”。进一步做好企业资金链断裂风险防范和处置，保障区域金融安全。健全防灾减灾救灾体制，实施《防汛防台公共规则》，加快应急指挥体系建设。落实企业安全生产主体责任，深化安全生产隐患排查治理，强化消防、渔船、道路交通安全突出问题专项整治，确保安全生产三项控制指标零增长。

　　大力支持驻台部队建设，做好国防教育、国防动员、民兵预备役和海防、人防工作，深化“双拥”工作，再创省级双拥模范城。

　　各位代表，我们将围绕人民群众最关心、最直接、最现实的利益问题，办好十方面民生实事。一是全面开展臭河、黑河、垃圾河整治，消灭垃圾河，改善臭河、黑河水质。完成农村生活污水治理专项规划编制，实施和提升800个村污水治理。二是城乡居民饮用水卫生监测合格率提高5个百分点。三是实施规范化市场建设三年行动计划，9家城镇农贸市场达标。四是大力发展公共交通，公交分担率提高3个百分点。五是县市区按常住人口数量等设立医疗救助专项资金，筹资水平达到人均13元以上。六是城乡居民最低生活保障月标准分别提高10%、13%;二档最低工资标准分别提高到1440元、1320元。七是开工建设9家健康养老机构，建成60个城镇社区居家养老服务中心，新增机构养老床位2800张。八是开工建设9个残疾人托养服务中心。九是对35岁至59岁的农村妇女实施“两癌”免费筛查。十是信息化村邮站新建1221个农村综合信息LED发布屏。

　　三、加强政府自身建设

　　新形势、新任务对政府工作提出更高要求，我们一定满怀豪情干事创业，义无反顾担当发展重任，清正廉洁树立良好形象。

　　推动新一轮思想大解放。坚持以思想解放引领改革创新，牢固树立改革意识、市场意识、担当意识，坚决消除阻碍发展的思维定势，坚决突破利益固化的藩篱，坚决革除体制机制的弊端，大胆探索，先行先试，争当思想解放先锋，争做改革开放试验田，唱响主旋律、发出好声音、凝聚正能量、营造好氛围。

　　提高依法行政能力。牢固树立法治理念，切实维护法律权威，促进社会公平正义。认真落实市人大及其常委会决议决定，自觉接受人大依法监督，积极支持人民政协履行政治协商、民主监督、参政议政职能，认真办理人大代表议案建议和政协提案。积极听取民主党派、人民团体和各界人士的意见建议，主动接受司法监督和社会公众监督。强化政府层级监督，加强监察、审计专门监督。健全民主决策机制，完善行政决策程序，加强重要政策跟踪评估，注重发挥决策咨询、法律顾问等专家作用。加强规范性文件和政府合同的监督管理。规范行政执法裁量权，加强行政复议规范化建设。建立和落实权力清单制度，深化行政权力规范化建设，构建网上权力运行体系，加快电子政务建设，提高政府工作透明度。

　　健全作风建设常态化制度。深化“以实干论英雄”工作竞赛活动，真抓实干、克难攻坚，比学赶超、争创一流。严格执行中央“八项规定”和省市委的具体要求，开展党的群众路线教育实践活动，贯彻“照镜子、正衣冠、洗洗澡、治治病”的总要求，坚决纠正形式主义、官僚主义、享乐主义和奢靡之风，着力解决群众反映的突出问题。全面落实中央厉行节约反对浪费有关要求，严格执行因公出国、公务接待、公务用车、办公用房、精减会议文件、“三公”经费管理等各方面规定。认真落实党风廉政建设责任制和廉政准则，推进反腐倡廉制度建设，健全廉政风险防控机制，强化对行政审批、招投标、土地出让、政府采购、公共资源交易等重点领域和关键环节的监管，严肃查处违法违纪案件，坚决防止和纠正损害群众利益的不正之风，努力建设人民满意政府。

　　各位代表，改革开创未来，实干成就梦想。让我们紧密团结在以习近平同志为总书记的党中央周围，在中共台州市委的坚强领导下，锐意进取，奋力前行，大干实干，干出精彩，为建设“山海秀丽、富裕和谐”的新台州而努力奋斗!
